# Supplementary material for: Impact of Combined Heat and Salt Stresses on Tomato Plants—Insights into Nutrient Uptake and Redox Homeostasis
Source: Antioxidants (Basel). 2022 Feb 28;11(3):478. doi: 10.3390/antiox11030478 (PMC8944476; doi:10.3390/antiox11030478)
Supplement: Supplementary file 1 [file antioxidants-11-00478-s001.zip › antioxidants-1591844-supplementary.pdf]

Supplementary Material

Table S1. Physicochemical characteristics of the Siro Royal universal substrate (SIRO©, Portugal) used for the plant assay.

**PHYSICOCHEMICAL CHARACTERISTICS**	
pH ($CaCl_2$)	5.5 – 6.5
Electric conductivity	50 – 100 µS cm^{-1}
Granulometry	0 – 15 mm
Organic matter	> 70%
NPK	19 – 7 – 10

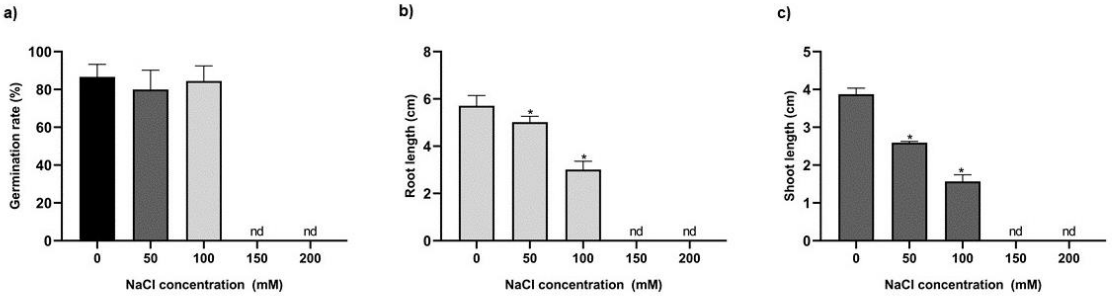

Figure S1. Percentage of germination (a), root length (b) and shoot length (c) of tomato seedlings grown for 7 days in solid MS nutritive medium, supplemented with different concentrations of NaCl (0, 50, 100, 150 and 200 mM). Data presented are mean ± SEM (n ≥ 3). Asterisks above the error bars indicate significant statistical differences between treatments and the control at $p \leq 0.05$, assessed through Tukey post-hoc test, following a one-way ANOVA.

Table S2. Results of the two-way ANOVA for all evaluated parameters in roots of *Solanum lycopersicum* L. var. *cerasiforme* after 21 days of exposure to 42 °C (4 hours per day) and irrigation with (100 mM) or without NaCl. Parameters where significant differences ($p \leq 0.05$) were recorded are highlighted in bold.

**PARAMETER (roots)**	**Factors**		**Interaction**
	SALT	***HEAT***	
**Length**	**F (1, 43) = 28.26; *p* < 0.0001**	**F (1, 43) = 52.66; *p* < 0.0001**	F (1, 43) = 0.6012; *p* = 0.4424
**Dry weight**	**F (1, 43) = 36.84; *p* < 0.0001**	**F (1, 43) = 40.38; *p* < 0.0001**	F (1, 43) = 0.5333; *p* = 0.4692
**Water content**	F (1, 19) = 0.4653; *p* = 0.4653	F (1, 19) = 0.9359; *p* = 0.3455	F (1, 19) = 0.007945; *p* = 0.9299
**Na^+**	**F (1, 8) = 656421; *p* < 0.0001**	**F (1, 8) = 2863; *p* < 0.0001**	**F (1, 8) = 1834; *p* < 0.0001**
**K^+**	**F (1, 8) = 19837; *p* < 0.0001**	**F (1, 8) = 691.1; *p* < 0.0001**	**F (1, 8) = 2244; *p* < 0.0001**
**Ca^{2+}**	**F (1, 8) = 3321; *p* < 0.0001**	**F (1, 8) = 55.13; *p* < 0.0001**	**F (1, 8) = 496.1; *p* < 0.0001**
**Mg^{2+}**	**F (1, 8) = 165.7; *p* < 0.0001**	**F (1, 8) = 414.0; *p* < 0.0001**	**F (1, 8) = 374.4; *p* < 0.0001**
**$O_2^{\cdot -}$**	F (1, 8) = 2.350; *p* = 0.1638	F (1, 8) = 0.0004534; *p* = 0.9835	F (1, 8) = 1.930; *p* = 0.2022
**H_2O_2**	F (1, 8) = 1.694; *p* = 0.2293	**F (1, 8) = 61.16; *p* < 0.0001**	F (1,8) = 1.731; *p* = 0.2248
**MDA**	**F (1, 11) = 5.810; *p* = 0.0346**	F (1, 11) = 4.635; *p* = 0.0544	F (1, 11) = 0.2805; *p* = 0.6069

**Proline**	**F (1, 9) = 830.0; *p* < 0.0001**	**F (1, 9) = 19.13; *p* = 0.018**	**F (1, 9) = 18.55; *p* = 0.0020**
**Total AsA**	F (1, 10) = 0.02314; *p* = 0.8821	F (1, 10) = 2.693; *p* = 1318	F (1, 10) = 0.6917; *p* = 0.4250
**AsA**	F (1, 10) = 0.06414; *p* = 0.8052	F (1, 10) = 0.01829; *p* = 0.8951	F (1, 10) = 0.1727; *p* = 0.6865
**DHA**	F (1, 9) = 0.1271; *p* = 0.7296	F (1, 9) = 1.268; *p* = 0.2892	F (1, 9) = 0.3393; *p* = 0.5745
**AsA/DHA**	F (1, 9) = 0.7880; *p* = 0.3978	F (1, 9) = 1.967; *p* = 0.1944	F (1, 9) = 0.1392; *p* = 0.7177
**GSH**	F (1, 9) = 2.005; *p* = 0.1905	**F (1, 9) = 18.68; *p* = 0.0019**	**F (1, 9) = 5.400; *p* = 0.0452**
**Total thiols**	F (1, 8) = 3.618; *p* = 0.0937	**F (1, 8) = 79.12; *p* < 0.0001**	F (1, 8) = 1.373; *p* = 0.2749
**Non-protein/ Protein thiols**	F (1, 10) = 0.1765; *p* = 0.6833	**F (1, 10) = 6.380; *p* = 0.0301**	F (1, 10) = 1.509; *p* = 0.2474
**SOD**	F (1, 11) = 0.006482; *p* = 0.9373	F (1, 11) = 2.057; *p* = 0.1793	F (1, 11) = 4.219; *p* = 0.0645
**CAT**	**F (1, 10) = 15.01; *p* = 0.0031**	**F (1, 10) = 9.137; *p* = 0.0128**	**F (1, 10) = 7.859; *p* = 0.0187**
**APX**	**F (1, 9) = 8.434; *p* = 0.0175**	**F (1, 9) = 43.25; *p* = 0.0001**	F (1, 9) = 0.001389; *p* = 0.9711
**DHAR**	**F (1, 9) = 7.860; *p* = 0.0206**	**F (1, 9) = 58.39; *p* < 0.0001**	**F (1, 9) = 24.81; *p* = 0.0008**
**GR**	F (1, 9) = 2.326; *p* = 0.1616	**F (1, 9) = 13.90; *p* = 0.0047**	**F (1, 9) = 34.27; *p* = 0.0002**

Table S3. Results of the two-way ANOVA for all evaluated parameters in shoots of *Solanum lycopersicum* L. var. *cerasiforme* after 21 days of exposure to 42 °C (4 hours per day) and irrigation with (100 mM) or without NaCl. Parameters where significant differences ($p \leq 0.05$) were recorded are highlighted in bold.

**PARAMETER (shoots)**	**Factors**		**Interaction**
	SALT	***HEAT***	
**Length**	**F (1, 36) = 107.0; *p* < 0.0001**	**F (1, 36) = 112.6; *p* < 0.0001**	F (1, 36) = 0.8989; *p* = 0.3494
**Dry weight**	**F (1, 42) = 59.36; *p* < 0.0001**	**F (1, 42) = 76.05; *p* < 0.0001**	F (1, 42) = 1.909; *p* = 0.1743
**Water content**	**F (1, 19) = 56.06; *p* < 0.0001**	**F (1, 19) = 6.082; *p* = 0.0233**	F (1, 19) = 0.1518; *p* = 0.7011
**Na^+**	**F (1, 8) = 1381045; *p* < 0.0001**	**F (1, 8) = 82240; *p* < 0.0001**	**F (1, 8) = 107573; *p* < 0.0001**
**K^+**	**F (1, 8) = 22713; *p* < 0.0001**	**F (1, 8) = 3027; *p* < 0.0001**	**F (1, 8) = 925.1; *p* < 0.0001**
**Ca^{2+}**	**F (1, 8) = 570.2; *p* < 0.0001**	**F (1, 8) = 44.18; *p* = 0.0002**	**F (1, 8) = 2424; *p* < 0.0001**
**Mg^{2+}**	**F (1, 8) = 19.00; *p* < 0.0024**	**F (1, 8) = 343.6; *p* < 0.0001**	**F (1, 8) = 104.0; *p* < 0.0001**
**$O_2^{\cdot-}$**	**F (1, 8) = 78.00; *p* < 0.0001**	**F (1, 8) = 16.65; *p* = 0.0035**	**F (1, 8) = 16.59; *p* = 0.0036**
**H_2O_2**	**F (1, 9) = 42.32; *p* = 0.0001**	**F (1, 9) = 38.81; *p* = 0.0002**	**F (1, 9) = 26.58; *p* = 0.0006**
**MDA**	**F (1, 9) = 57.70; *p* < 0.0001**	**F (1, 9) = 47.30; *p* < 0.0001**	**F (1, 9) = 19.71; *p* = 0.0016**
**Proline**	**F (1, 9) = 408.4; *p* < 0.0001**	**F (1, 9) = 26.47; *p* = 0.0006**	**F (1, 9) = 22.06; *p* = 0.0011**
**Total AsA**	**F (1, 9) = 7.639; *p* = 0.0220**	F (1, 9) = 3.930; *p* = 0.0788	F (1, 9) = 2.925; *p* = 0.1214
**AsA**	F (1, 9) = 0.5398; *p* = 0.4812	**F (1, 9) = 6.613; *p* = 0.0301**	F (1, 9) = 1.180; *p* = 0.3056
**DHA**	**F (1, 9) = 19.01; *p* = 0.0018**	F (1, 9) = 1.573; *p* = 0.2414	F (1, 9) = 4.470; *p* = 0.0636
**AsA/DHA**	**F (1, 10) = 5.243; *p* = 0.0450**	**F (1, 10) = 11.76; *p* = 0.0064**	F (1, 10) = 0.004423; *p* = 0.9483
**GSH**	**F (1, 10) = 32.78; *p* = 0.0002**	F (1, 10) = 0.002158; *p* = 0.9639	**F (1, 10) = 20.78; *p* = 0.0010**
**Total thiols**	**F (1, 11) = 8.443; *p* = 0.0143**	F (1, 11) = 1.035; *p* = 0.3309	**F (1, 11) = 16.34; *p* = 0.0019**
**Non-protein/ Protein thiols**	**F (1, 10) = 52.82; *p* < 0.0001**	**F (1, 10) = 58.90; *p* < 0.0001**	**F (1, 10) = 34.17; *p* = 0.0002**
**SOD**	**F (1, 10) = 61.00; *p* < 0.0001**	**F (1, 10) = 9.535; *p* = 0.0115**	**F (1, 10) = 20.44; *p* = 0.0011**
**CAT**	**F (1, 11) = 16.66; *p* = 0.0018**	**F (1, 11) = 30.00; *p* = 0.0002**	F (1, 11) = 1.695; *p* = 0.2196
**APX**	**F (1, 8) = 6.117; *p* = 0.0385**	**F (1, 8) = 25.41; *p* = 0.0010**	**F (1, 8) = 15.42; *p* = 0.0044**
**DHAR**	**F (1, 10) = 40.70; *p* < 0.0001**	**F (1, 10) = 6.185; *p* = 0.0322**	**F (1, 10) = 21.95; *p* = 0.0009**
**GR**	**F (1, 11) = 58.88; *p* < 0.0001**	**F (1, 11) = 6.330; *p* = 0.0287**	F (1, 11) = 1.646; *p* = 0.2259

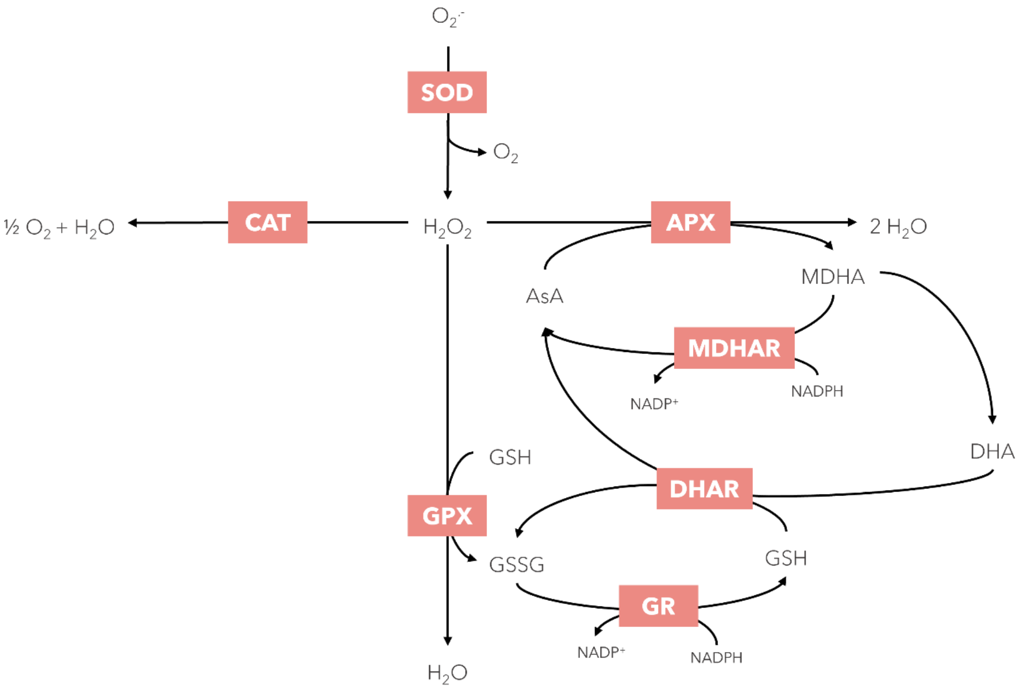

Figure S2. Enzymatic pathways that regulate redox status in plants. Superoxide anion ($O2^{\cdot -}$), hydrogen peroxide (H_2O_2), superoxide dismutase (SOD), catalase (CAT), ascorbate peroxidase (APX), monodehydroascorbate reductase (MDHAR), dehydroascorbate reductase (DHAR), glutathione reductase (GR), glutathione peroxidases (GPX), ascorbate (AsA), monodehydroascorbate (MDHA), dehydroascorbate (DHA), oxidized glutathione (GSH), reduced glutathione (GSSG).
